# Characterization of the quinol-dependent nitric oxide reductase from the pathogen *Neisseria meningitidis*, an electrogenic enzyme

**DOI:** 10.1038/s41598-018-21804-0

**Published:** 2018-02-26

**Authors:** Nathalie Gonska, David Young, Riki Yuki, Takuya Okamoto, Tamao Hisano, Svetlana Antonyuk, S. Samar Hasnain, Kazumasa Muramoto, Yoshitsugu Shiro, Takehiko Tosha, Pia Ädelroth

**Affiliations:** 1 Department of Biochemistry and Biophysics, Stockholm University, Svante Arrhenius väg 16C, 10691 Stockholm, Sweden; 2 RIKEN SPring-8 Center, 1-1-1 Kouto, Sayo, Hyogo, 679-5148 Japan; 3 Molecular Biophysics Group, Institute of Integrative Biology, Faculty of Health and Life Sciences, University of Liverpool, Liverpool, L69 7ZB UK; 4 Graduate School of Life Science, University of Hyogo, 3-2-1 Kouto, Kamigori, Ako, Hyogo, 678-1297 Japan; 5 Present Address: Center for Structural Biology, VIB, B-1050 Brussels, Belgium

## Abstract

Bacterial nitric oxide reductases (NORs) catalyse the reduction of NO to N_2_O and H_2_O. NORs are found either in denitrification chains, or in pathogens where their primary role is detoxification of NO produced by the immune defense of the host. Although NORs belong to the heme-copper oxidase superfamily, comprising proton-pumping O_2_-reducing enzymes, the best studied NORs, *c*NORs (cytochrome *c*-dependent), are non-electrogenic. Here, we focus on another type of NOR, qNOR (quinol-dependent). Recombinant qNOR from *Neisseria meningitidis*, a human pathogen, purified from *Escherichia coli*, showed high catalytic activity and spectroscopic properties largely similar to *c*NORs. However, in contrast to *c*NOR, liposome-reconstituted qNOR showed respiratory control ratios above two, indicating that NO reduction by qNOR was electrogenic. Further, we determined a 4.5 Å crystal structure of the *N. meningitidis* qNOR, allowing exploration of a potential proton transfer pathway from the cytoplasm by mutagenesis. Most mutations had little effect on the activity, however the E-498 variants were largely inactive, while the corresponding substitution in *c*NOR was previously shown not to induce significant effects. We thus suggest that, contrary to *c*NOR, the *N. meningitidis* qNOR uses cytoplasmic protons for NO reduction. Our results allow possible routes for protons to be discussed.

## Introduction

Bacterial nitric oxide reductases (NOR) are membrane-integrated enzymes that reduce nitric oxide (NO) to nitrous oxide (N_2_O) according to Eq. 1

1 $$2{\rm{NO}}+2{{\rm{H}}}^{+}+2{{\rm{e}}}^{-}\to {{\rm{N}}}_{2}{\rm{O}}+{{\rm{H}}}_{2}{\rm{O}}$$

NORs are typically found in anaerobic respiration that uses nitrate as terminal electron acceptor, termed denitrification. However, some NORs are found in pathogenic bacteria where they protect against toxic NO produced by macrophages in the host’s immune system.

NORs are members of the heme-copper oxidase (HCuO) superfamily and are usually further subdivided into cytochrome *c*-oxidising *c*NORs and quinol-oxidising qNORs (for a recent review, see^1^). *c*NORs are purified as a complex of NorB, the subunit harboring the binuclear heme *b*_3_–non heme iron (Fe_B_) active site, and NorC with an electron-accepting heme *c*. qNORs are single-subunit (termed NorZ) enzymes. In NorZ, the C-terminal part is homologous to NorB and the N-terminal to NorC, where the N-terminal retains the cytochrome *c* fold although the heme *c* is absent.

O_2_-reducing HCuOs of all major types (A, B and C) have been shown to conserve energy from O_2_-reduction by creating a proton electrochemical gradient across the membrane (for classification and reviews on HCuOs, see^2–4^). This is achieved in two ways; first protons needed for O_2_ reduction originate exclusively from the negative inside. In addition protons are pumped across the membrane. In sharp contrast, for the best characterised NORs, *c*NORs, the catalytic reaction is non-electrogenic^5–7^. This may seem surprising since NO-reduction is, just like O_2_-reduction, highly exergonic. Recently, however, the NOR from *Bacillus azotoformans*, Cu_A_NOR, which is homologous to the B-type HCuOs^8,9^, and is more distantly related to both *c-* and qNORs, was reported to indeed be electrogenic^9^. In *c*NOR, the lack of electrogenicity means that no protons are pumped, and that protons needed for the reduction of NO (Eq. 1) are taken up from the periplasmic solution. These properties are consistent with the crystal structure of *c*NOR from *Pseudomonas aeruginosa*^10^ and the mutational analysis of *P. denitrificans c*NOR^11–14^.

For qNORs, there is, to our knowledge, no published study on whether they are electrogenic. In the crystal structure of the qNOR from *Geobacillus stearothermophilus* (*Gs*qNOR), there is a water-filled channel lined with charged and/or polar residues that connects the active site with the cytoplasmic side^15^. If this pathway is used for proton transfer during NO reduction, the enzyme would be electrogenic. Unfortunately, the NO reduction activity of the *Gs*qNOR was very low, presumably due to the high fraction of the enzyme in which the active site Fe_B_ was replaced by catalytically inert zinc^15^, which hampered further functional analysis.

In this study, to obtain a more active qNOR for functional studies, we focussed on pathogens that utilize qNOR for detoxification of NO, and chose *Neisseria meningitidis*, a gram-negative β-proteobacterium, which in its virulent form causes meningitis and septicaemia. In *N. meningitidis*, the qNOR is responsible for survival of the bacterial colonies within macrophages^16^. The *N. meningitidis* qNOR *(Nm*qNOR) shows 36% sequence identity to the *Gs*qNOR, and functionally important residues are well-conserved between them (Supplementary Fig. S1). Recombinant *Nm*qNOR, expressed and purified from *E. coli*, showed an NO reduction activity of ~30 e^−^/s/qNOR, about an order of magnitude higher than for any previously reported qNOR^15,17,18^. We determined a 4.5 Å resolution X-ray structure for *Nm*qNOR that shows close similarity to the *Gs*qNOR^15^. Using liposome-reconstituted qNOR, we present evidence that the enzyme creates a proton electrochemical gradient during NO turnover. Possible proton pathways were explored by functional studies of a number of variants constructed by site-directed mutagenesis.

## Results

### Expression and purification of recombinant *N. meningitidis* qNOR

The expression of the active form of *Nm*qNOR in *E. coli* was monitored by measuring the NO reduction activity in the isolated membranes, since *E. coli* has no membrane proteins with significant NO reduction activity. This way, we found that the best expression condition was obtained with the C43 (DE3) strain cultured at 37 °C (without any supplements) and by induction of expression with IPTG at OD_600_ = 0.5. The isolated membranes showed an NO consumption activity of 7–10 nmol/s/mg protein, whereas the corresponding activity in membranes isolated from C43 without *Nm*qNOR expressed was less than 0.01 nmol/s/mg protein.

**Figure 1:**
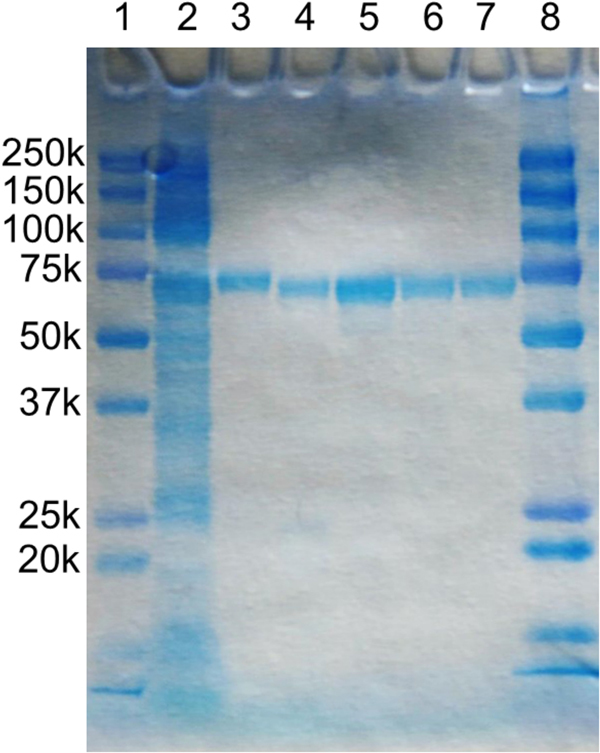
SDS-PAGE of *N. meningitidis* qNOR samples during purification. Conditions: c-PAGEL (Atto corporation), 12.5% gel was used with Tris-glycine running buffer. Lanes 1 and 8: marker with molecular sizes as indicated, 2: solubilized membranes, 3: Ni-NTA purified qNOR, 4: HIS-tag cleaved qNOR, 5–7: SEC fractions.

The solubilization and purification procedures were also optimized by monitoring the NO reduction activity. Membranes were solubilised with 1% dodecyl-β-D-maltoside (DDM) and active *Nm*qNOR purified in the presence of 0.05% DDM by Ni affinity (IMAC) and size exclusion (SEC) chromatography, giving a preparation that shows a single band (at ~70 kDa) upon SDS-PAGE (Fig. 1). The ratio of the absorbance at 408 nm (heme) to the absorbance at 280 nm (total protein) A_408_/A_280_ was ~0.7, which is comparable to that of the *Gs*qNOR preparation used for crystallization^15^. The yield after IMAC purification was ~3 mg qNOR/L (culture). The purified enzyme showed an NO consumption activity of ~300 nmol/s/mg protein.

### Metal content and spectroscopic characterization of *N. meningitidis* qNOR

The purified wild-type *Nm*qNOR contained 2.7 ± 0.2 equivalents of iron, 0.2 ± 0.1 equivalents of Zn and negligible Cu. This indicates the presence of the two hemes *b* (one low-spin heme *b* and one high-spin heme *b*_3_ in the active site) and that the Fe_B_ site is mostly occupied by iron.

**Figure 2:**
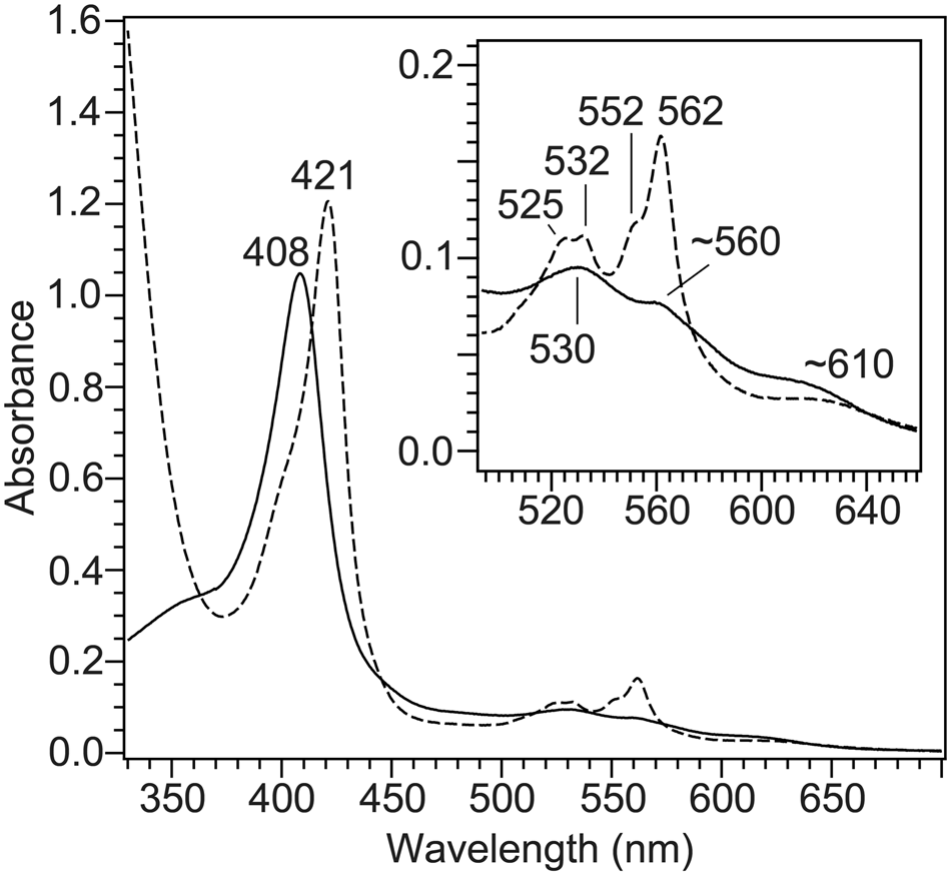
Visible absorption spectra of wild-type *N. meningitidis* qNOR. The spectra shown are oxidized state (solid curve) and dithionite-reduced state (broken curve). The sample is in 50 mM Tris-HCl pH 8.0, 150 mM NaCl, and 0.1% DM. The reduced qNOR sample was prepared by the addition of an excess amount of dithionite to the oxidized enzyme under N_2_ atmosphere.

Figure 2 displays optical absorption spectra of the oxidized and reduced *Nm*qNOR. The Soret peak at 408 nm and Q-band at 530 and ~560 nm in the oxidized state are similar to those in *Gs*qNOR. In addition, a broad absorbance band around 600 nm is assigned to a charge transfer band of the high-spin heme *b*_3_. Upon reduction by dithionite, the Soret peak shifted to 421 nm, and four peaks were observed in the Q-band region at 525, 532, ~552 and 562 nm (Fig. 2). Given that a ferrous low-spin heme *b* generally gives only one major peak at 560 nm with a smaller peak at ~530 nm, it is likely that these four peaks arise from two subpopulations with the heme *b* in slightly different environments.

**Figure 3:**
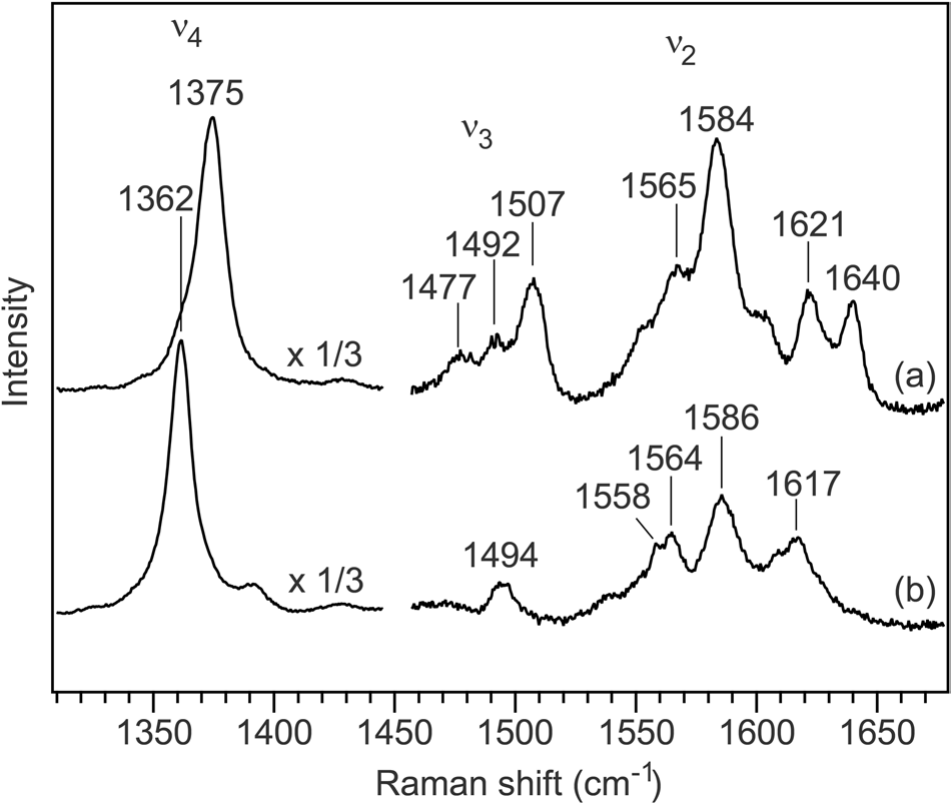
High frequency region of resonance Raman spectra of wild-type qNOR from *N. meningitidis*. Traces shown are (**a**) oxidized and (**b**) dithionite-reduced qNOR. The spectra were obtained with excitation at 413.1 nm. The qNOR concentration was adjusted to 20–40 µM in 50 mM Tris-HCl pH 8.0, 150 mM NaCl, and 0.1% DM. The reduced form was prepared by addition of an excess amount of dithionite under N_2_ atmosphere.

Resonance Raman spectra of *Nm*qNOR were acquired to further probe the heme structural properties. Upon excitation at the Soret region (413.1 nm), we obtained data presented in Fig. 3 in the high frequency region, which contains rich information on the coordination and electronic structures of the hemes. In the oxidized state, a coordination and spin state marker, ν_3_ lines, were detected at 1477, 1492 and 1507 cm^−1^, which can be assigned to a 6-coordinated high-spin, a 5-coordinated high-spin and a 6-coordinated low-spin heme, respectively^19^. The high-spin heme signal is consistent with the charge transfer band in the absorption spectrum. An oxidation state ν_4_ marker line was shifted from 1375 cm^−1^ to 1362 cm^−1^ by the addition of dithionite, confirming complete reduction of both hemes. In contrast to the oxidized state, a single ν_3_ line observed at 1494 cm^−1^ indicates that in the reduced state, both hemes mainly adopt a 6-coordinate low-spin state.

### NO and O_2_ reduction activity in *N. meningitidis* qNOR

**Figure 4:**
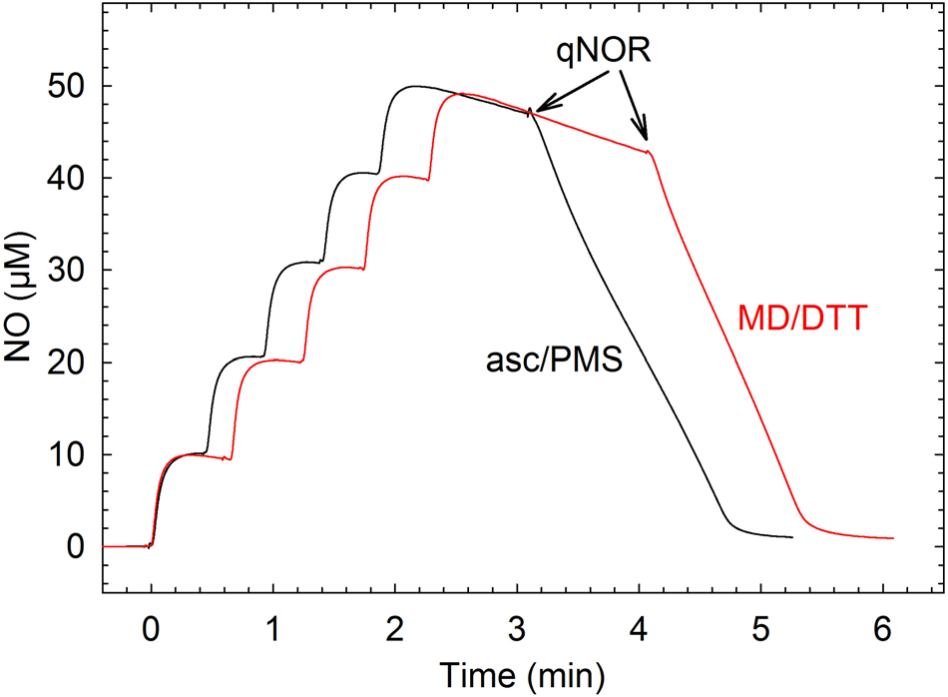
NO-reduction activity of *N. meningitidis* qNOR with MD/DTT (1 mM/5 mM, red trace) and PMS/Asc (10 µM/6 mM, black trace). Conditions: 20 mM K^+^-HEPES (pH 7.4), 100 mM KCl, 0.05% DDM, 10 mM glucose, 100 U/mL catalase, 10 U/mL glucose oxidase. About 5 times higher concentration of qNOR was used with PMS/Asc (~70 nM; black trace, compared to ~14 nM; red trace). NO was added in 5 consecutive steps (in total 50 µM); all additions except qNOR (addition indicated) were made before adding NO.

The purified *Nm*qNOR showed an NO reduction activity of 30 ± 9 NO/s/qNOR (30 e^−^/s/qNOR) with the menadione (MD)/dithiothreitol (DTT) reduction system (Fig. 4, Table 1). The activity was lower with the phenazine methosulfate (PMS)/ascorbate (Asc) system under the conditions used in Table 1. The values given are the maximum activities, which occurred at ~5 μM NO, as there is a sigmoidal behavior in the NO consumption curve, indicative of substrate inhibition at high [NO] (see Fig. 4). Substrate inhibition, also observed for *c*NOR^20^, is seen with both the MD/DTT and PMS/Asc electron donor systems, but less prominent when higher concentrations of reductant/mediator were used (as observed also for *c*NOR^21^). The NO-reduction activity is pH dependent with a maximum at pH 7.0–7.5 (Supplementary Fig. S2).

**Table 1:** NO-reduction activity of wild-type *N. meningitidis* qNOR.

	detergent (e^−^s^−1^)	liposomes (e^−^s^−1^)	RCR
MD/DTT (1 mM/5 mM)	30 ± 10 (*n* = 12)	10.1 ± 0.4 (*n* = 3)	2.0 ± 0.1 (*n* = 4)
PMS/Asc (10 µM/6 mM)	5 ± 1 (*n* = 8)	5.4 ± 0.2 (*n* = 3)	2.0 ± 0.3 (*n* = 4)

Experimental conditions: 20 mM HEPES (pH 7.4), 100 mM KCl, (+0.05% DDM for the solubilized samples), 10 mM glucose, 100 U/mL catalase, 10 U/mL glucose oxidase. The activity for liposomes is in the presence of the uncoupler CCCP. For RCR calculations, see text. The numbers given are the averages and standard deviations for *n* repetitions as specified in Materials and Methods.

To test the selectivity of the electron donor in *Nm*qNOR, we assayed the activity also with a soluble donor, cytochrome *c* (cyt. *c*). No significant NO reduction activity (~3% of that with MD/DTT) was observed with the cyt. *c*/N,N,N’,N’-tetramethyl-phenylenediamine (TMPD)/Asc system. Addition of the redox-inactive quinone analogue, HQNO, to the assay solution inhibits the turnover activity both for the MD/DTT and Asc/PMS electron donation systems.

Furthermore, the *Nm*qNOR shows cross-reactivity with O_2_ as substrate with a turnover of 0.5 ± 0.1 O_2_/s/qNOR (corresponding to 2.0 ± 0.4 e^−^/s/qNOR, assuming that O_2_ is reduced to H_2_O as in *c*NOR^22^) using the MD/DTT system, similar to that observed for the *Gs*qNOR (3 e^−^/s/qNOR)^23^.

### Activity and RCR of *N. meningitidis* qNOR in liposomes

The uncoupled (in the presence of the protonophore carbonyl cyanide m-chlorophenyl hydrazone (CCCP)) NO reduction activity of liposome-reconstituted *Nm*qNOR was 10.1 ± 0.2 e^−^/s/qNOR (~35% of that observed in detergent-solubilized qNOR) using the MD/DTT donor system. With the PMS/Asc (10 µM/6 mM) donor system, the activity was 5.4 ± 0.2 e^−^/s/qNOR which corresponds to ~100% of the solubilized sample (Table 1). These relative differences are possibly related to the solubility of the electron donors/mediators in the membrane versus detergent, or differences in how well the donor system can access the two qNOR populations (oriented ‘inside-out’ or ‘right-side out’) in the liposomes.

**Figure 5:**
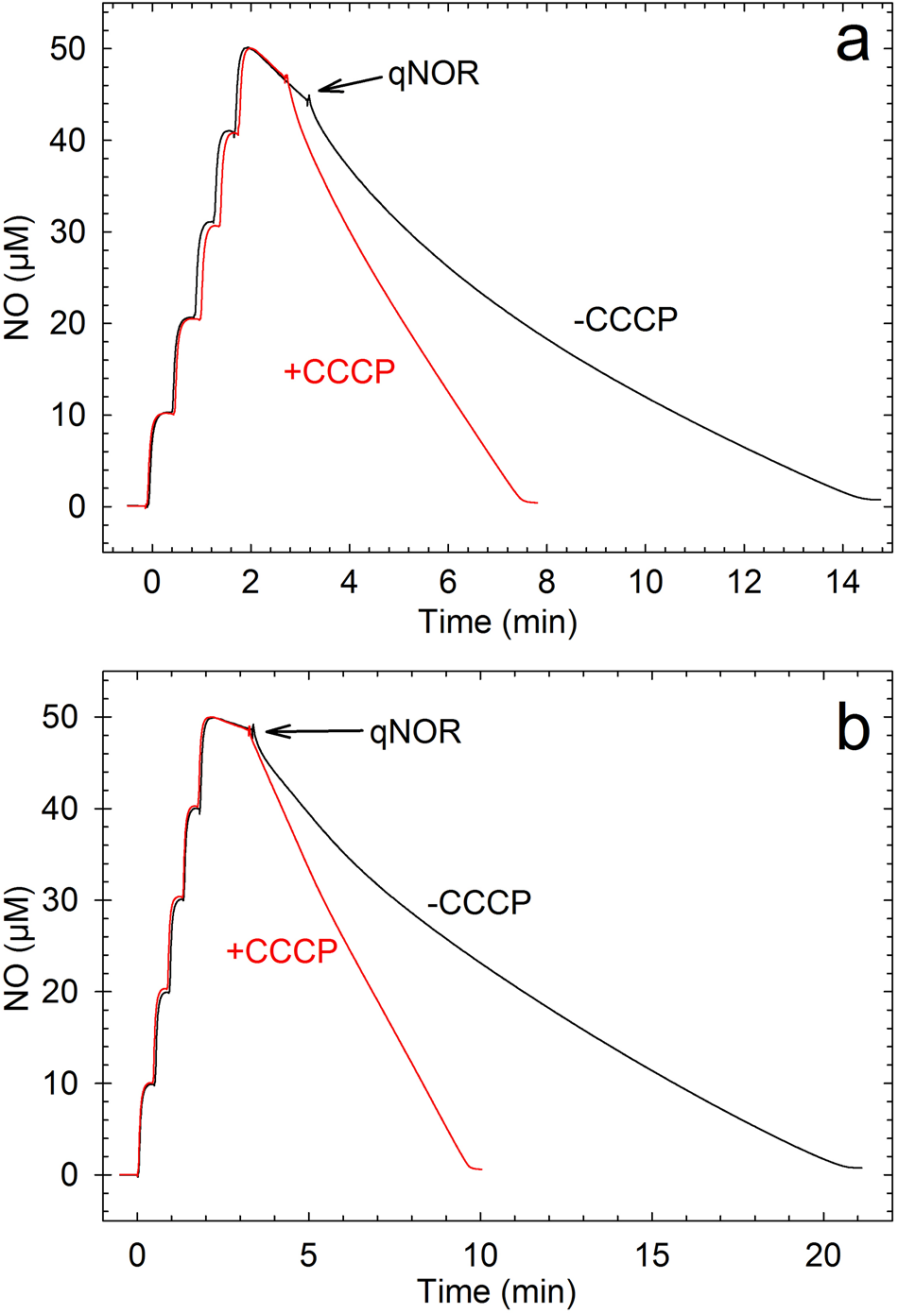
The effect of uncouplers on the activity of qNOR in liposomes. a: With MD/DTT and b: with Asc/PMS. Conditions: 20 mM K^+^-HEPES (pH 7.4), 100 mM KCl, 10 mM glucose, 100 U/mL catalase, 10 U/mL glucose oxidase, (+10 µM CCCP for red traces). All additions except qNOR were made before NO addition. qNOR in liposomes (40 nM) was added where indicated.

Liposome-reconstituted *Nm*qNOR is thus highly active, allowing further analysis. The ratio of the catalytic turnover rates observed in the presence (‘uncoupled’) and absence (‘coupled’) of ionophors and/or protonophors (as CCCP) is called the ‘respiratory control ratio’ (RCR). An RCR >1 indicates that there is formation of a membrane potential. *Nm*qNOR shows RCRs induced by CCCP of 2.0 ± 0.1 during NO reduction with both the MD/DTT and PMS/Asc donor-mediator systems (Table 1, Fig. 5). Addition of the K^+^-ionophore valinomycin (VAL) alone or together with CCCP also gave rise to RCRs with both electron-mediator-systems (Supplementary Table S1).

As control experiments, we compared the effect of uncouplers on the *Nm*qNOR activity to that for liposome-reconstituted *P. denitrificans c*NOR, which is known to use protons from the ‘outside’ periplasmic solution. For this comparison we used a ‘compromise’ pH and electron mediator system (PMS/Asc and pH 7) that gives about the same turnover rate for both enzymes. Under these identical conditions, *c*NOR in liposomes expectedly showed an RCR close to 1 (Supplementary Fig. S3, RCR is ~0.7), whereas *Nm*qNOR showed an RCR of 2.4. We note that, in contrast to *c*NOR, only uncoupled *Nm*qNOR-liposomes show substrate inhibition (Fig. 5 and Supplementary Fig. S3, see Discussion).

### Crystal structure of *N. meningitidis* qNOR at a resolution of 4.5 Å

**Figure 6:**
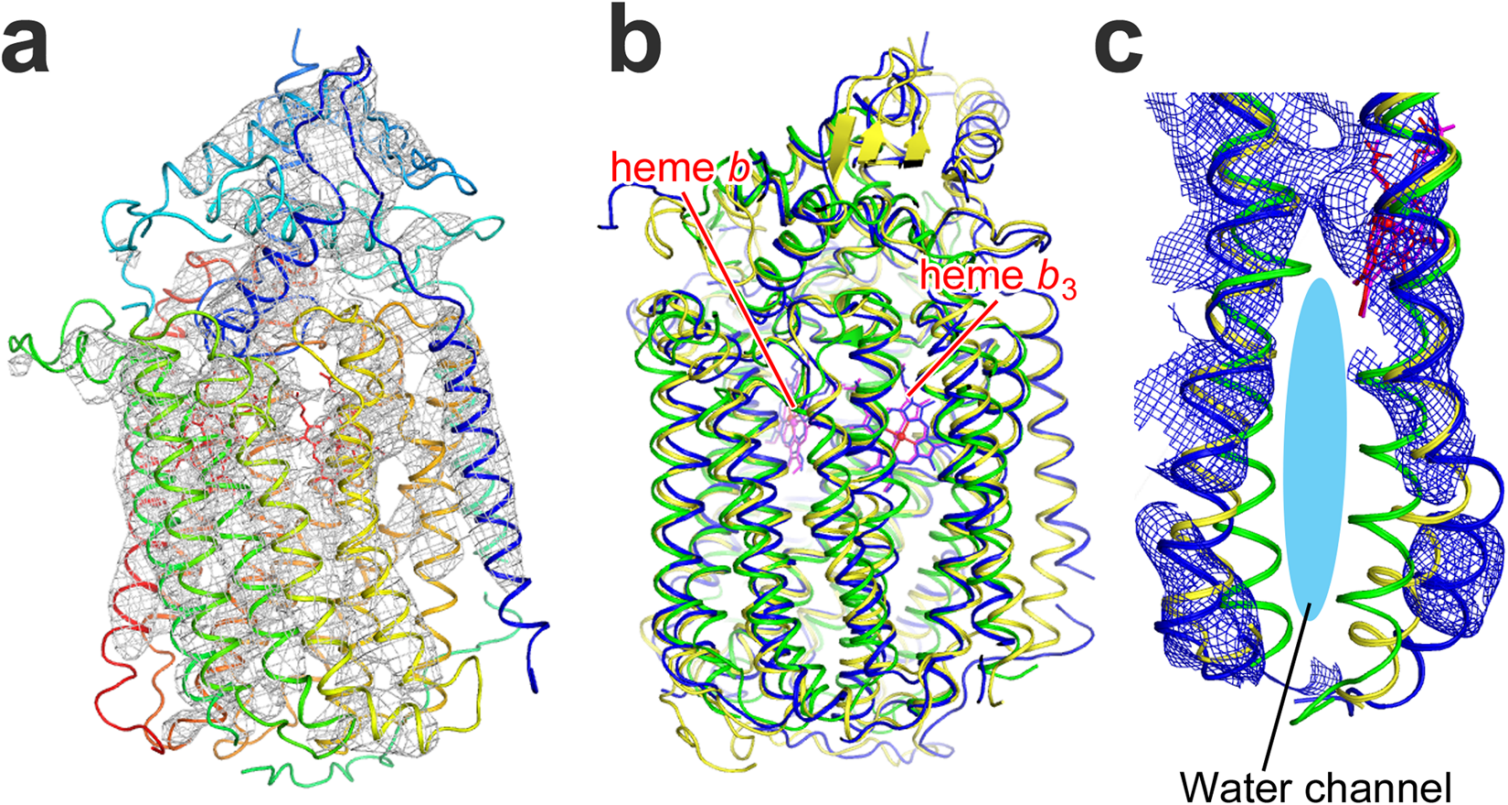
Crystal structure of *N. meningitidis* qNOR at a resolution 4.5 Å. (**a**) Overall structure with 2*F*_o_ – *F*_c_ electron density map contoured at 2.0σ (gray mesh). (**b**) Superposition of the Cα traces of *G. stearothermophilus* qNOR (PDB ID: 3AYF, yellow ribbon) and *P. aeruginosa c*NOR (PDB ID: 3O0R, green ribbon) onto that of *Nm*qNOR (blue ribbon). The root-mean-square deviations of the Cα atoms are 1.19 and 2.23 Å for *Nm*qNOR-*Gs*qNOR and *Nm*qNOR-*c*NOR, respectively. Heme *b* and *b*_3_ in *N. meningitidis*, *G. stearothermophilus* qNORs and *c*NOR are shown by red, magenta and blue sticks, respectively. (**c**) The water channel region in the superposed structure. The ribbon colors correspond to panel (b). Blue mesh represents a composite omit map of *Nm*qNOR contoured at 1.5σ.

The crystal structure of *Nm*qNOR was solved at a resolution of 4.5 Å (Fig. 6a). Despite the low resolution, the electron density map permitted us to trace the transmembrane helices. The *R*_work_/*R*_free_ values (0.324/0.359, see Supplementary Table S2 for details) of the model structure of *Nm*qNOR is within the acceptable range for structures of similar resolution, implying that the current structure is reliable. Although a high-resolution structure is definitely required for structural information on the active site of *Nm*qNOR, the overall structural fold aligns well to those of *Gs*qNOR and *c*NOR as shown in the superposed structure (Fig. 6b). However, when comparing the region of *Nm*qNOR that overlaps with the water channel region of *Gs*qNOR and the corresponding region of *c*NOR, the arrangement of the surrounding helices of *Nm*qNOR is more similar to that of *Gs*qNOR than that of *c*NOR (Fig. 6c). Thus, it is highly plausible that *Nm*qNOR has a water channel like *Gs*qNOR.

### Characterization of *N. meningitidis* qNOR variants of putative water channel residues

**Figure 7:**
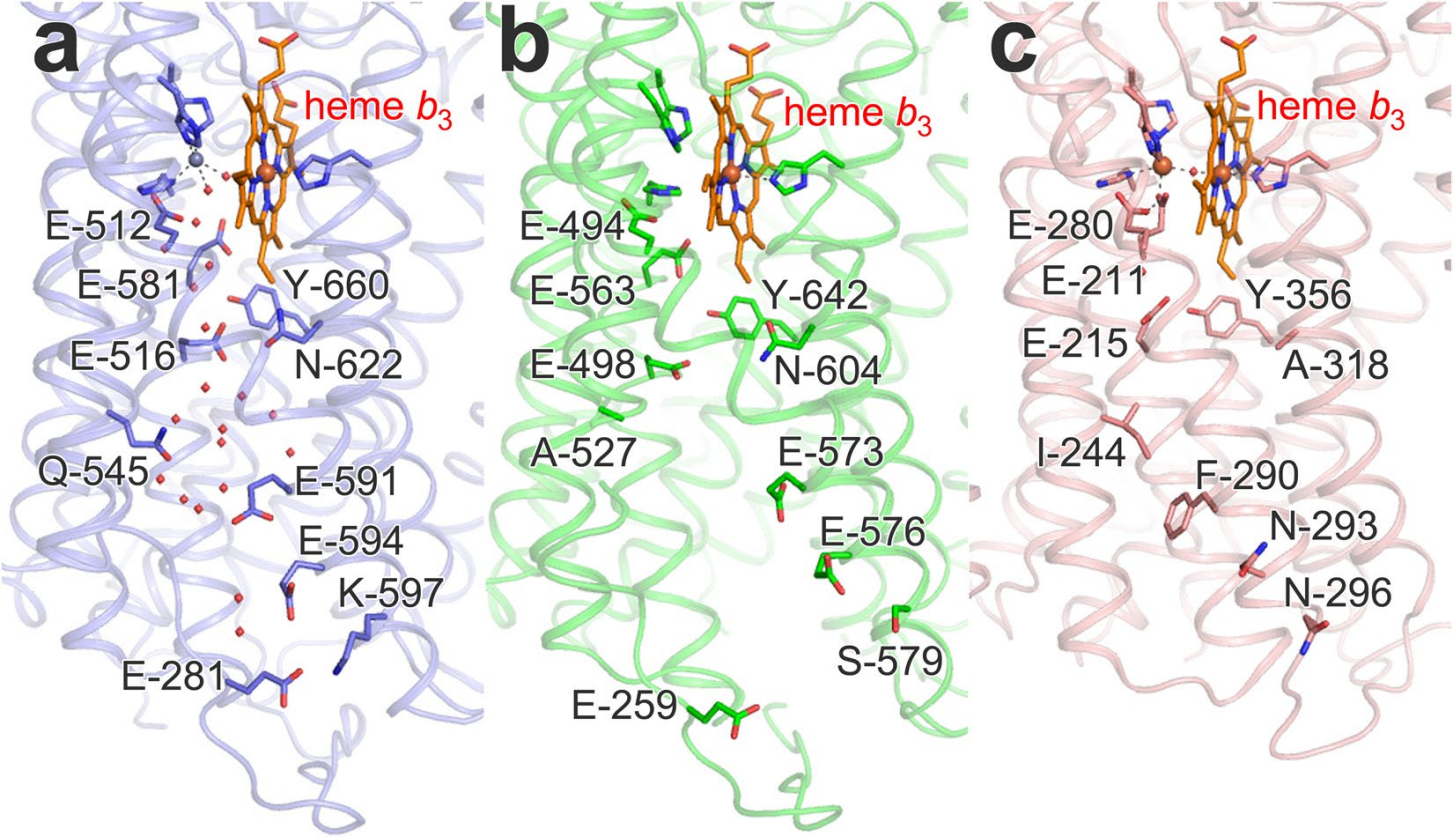
Structure of the water channel region in *G. stearothermophilus* qNOR (pdb ID 3AYF^15^) (**a**), the corresponding region in the low-resolution structure of *N. meningitidis* qNOR (**b**) compared also to *P. aeruginosa c*NOR (pdb ID 3O0R^10^) (**c**). Although the orientation of the side-chains of the amino acid residues could not be determined in the low resolution structure of *Nm*qNOR, the residues that correspond to those in *Gs*qNOR are modeled for positional comparison. Water molecules observed in the X-ray crystal structure of *Gs*qNOR are shown as red spheres. The amino acid alignment (Supplementary Fig. S1) shows that E-281 in *Gs*qNOR is replaced by a Thr (−255) in *Nm*qNOR, but there is a Glu (−259) in a corresponding spatial location. Note that the iron in the Fe_B_ site (dark red sphere) in the *c*NOR structure (c) is replaced by a Zn ion (grey sphere) in *Gs*qNOR, but that this site is presumably occupied by Fe in *Nm*qNOR as described in Results. Figure made using the Pymol program.

We compared the X-ray crystal structure of *Gs*qNOR^15^ with the 4.5 Å resolution structure of *Nm*qNOR, focusing on the possible conservation of the water-filled putative proton pathway that spans the distance between the cytosol and the active site in *Gs*qNOR (Figs 6 and 7). On the basis of this comparison, and attempting to block proton conduction, we made substitutions in *Nm*qNOR for E-259, located at the entrance, A-527 and E-573, the two latter being located further ‘up’ the water channel. As the E-281 residue (in *Gs*qNOR), at the entrance of the water-filled channel, shows poor conservation among qNOR sequences (see^24^ for conservation patterns of qNOR), we exchanged the E-259 in *Nm*qNOR which is in similar spatial location. E-573 is conserved to *Gs*qNOR (and other qNORs, see^24^), and A-527 is a glutamine (Gln-545, not highly conserved) in *Gs*qNOR (Fig. 7). These polar or protonatable residues are not conserved to *c*NOR, which instead contain hydrophobic residues at these locations (Fig. 7). Table 2 compiles the results obtained with the qNOR variants prepared. Unfortunately, the A527F variant (or any of the double variants containing it) did not express, possibly due to the bulky hydrophobic phenylalanine inducing too large a structural change. The other variants could be expressed and purified by the same procedures used for wild-type *Nm*qNOR.

**Table 2:** Properties of *N. meningitidis* qNOR wild-type and variants of putative water channel residues. Values given are the averages and standard deviations for *n* repetitions.

Variant	Expression level	NO consumption rate (% wild-type)	Non-heme iron content (% wild-type)
WT	+++	100^*b*^	100
A527F	−^*a*^	−^*a*^	n.d.
E259L/A527F	−^*a*^	−^*a*^	n.d.
E259Q/A527F	−^*a*^	−^*a*^	n.d.
E573F/A527F	−^*a*^	−^*a*^	n.d.
E259Q	+++	102 ± 5^*b*^	n.d.
E259L	+++	95 ± 6^*b*^	n.d.
E259Q/E573F	+++	70 ± 10^*b*^	n.d.
E259L/E573F	+	79 ± 7^*b*^/42 ± 4^*c*^	~130 (*n* = 1)
E498A	++++	7 ± 2^*c*^	35 ± 1 (*n* = 2)
E498Q	++++	20 ± 3^*c*^	30 ± 2 (*n* = 2)
E498F	+	1.5 ± 0.4^*c*^	100 ± 20 (*n* = 2)

^*a*^Variant showed no or very low expression in *E. coli*.

^*b*^Rate obtained using the PMS/Asc reduction system.

^*c*^Rate obtained using the MD/DTT reduction system.

The purified variants showed optical absorption spectra essentially identical to that of the wild-type, indicating that the amino acid substitutions did not seriously affect the active site structure. In addition, these variants retained NO reduction activity (40–100% of that of wild-type) as seen in Table 2.

We also exchanged the E-498 residue, which is conserved in the whole NOR family including *c*NORs (see Fig. 7 and Discussion). The E-498 variants all show low catalytic activity (1–20% of wild-type, see Table 2). For inactive qNOR variants, the non-heme iron content was assayed using the ferene method (Table 2), and significantly reduced levels were observed in the E498A (35%) and E498Q (30%) variants, while E498F variant retained ~100% non-heme iron.

The E259L/E573F double variant was reconstituted into liposomes and the RCR of NO reduction rates was determined. The uncoupled activity of the reconstituted E259L/E573F variant was 12.9 ± 0.5 e^−^/s/qNOR (100% of wild-type) and the RCR 1.2 ± 0.1. For the E-498 variants, we attempted to asses RCRs only for E498Q, which had the highest activity. We observed an RCR slightly above 1, but the activity was too low in liposomes to draw any conclusions about buildup of a gradient.

### Co-reconstitution of wild-type and E259L/E573F variant qNOR with cytochrome *aa*_3_ oxidase

Because we observed a smaller RCR with the E259L/E573F variant than with wild-type qNOR, we wanted to correct for possible differences in quality and tightness of the proteoliposomes, factors that can influence RCR values. Therefore, we co-reconstituted the wild-type and E259L/E573F variant qNORs together with the cytochrome *c* oxidase (cyt. *aa*_3_) from *Rhodobacter sphaeroides*, and used the RCR observed for O_2_-reduction by the latter as a ‘ruler’ for the quality of the liposomes (as done for *c*NOR previously^6^). The data obtained is presented in Supplementary Table S3 and shows that using the ratio of the RCR for NO over that for O_2_, there is no significant difference between the E259L/E573F variant (RCR_NO_/RCR_O2_ = 0.45) and wild-type qNOR (RCR_NO_/RCR_O2_ = 0.35).

## Discussion

The superfamily of HCuOs share a common evolutionary origin, but how to root the evolutionary tree is still an open question (see e.g.^25–27^). One intriguing difference between the O_2_-reducing HCuOs and the NORs is the difference in conservation of energy; whereas O_2_-reducing HCuOs conserve a large fraction of the energy available from O_2_-reduction in the form of a proton electrochemical gradient, the well-studied *c*NOR does not conserve the equally large free energy available from NO reduction, and is thus non-electrogenic^5–7^. Accordingly, the crystal structure of *c*NOR showed putative proton transfer pathways only from the periplasmic side, and the area from the catalytic site towards the cytoplasm is hydrophobic^10^. Site-directed mutagenesis studies are consistent with this, showing that protons transferred in *c*NOR use only one defined pathway originating in the periplasmic bulk^13,14^. When the structure of qNOR from *G. stearothermophilus* was determined, the most surprising feature was therefore the observation of a large water-filled cavity leading from the cytoplasmic bulk into the active site of the enzyme^15^ and overlapping in location with that of the K-pathways for proton transfer in the O_2_-reducing HCuOs^1^. The residues of the K-pathway are not conserved among the A, B and C-type oxidases but rather the spatial location is conserved^28,29^. If the *Gs*qNOR K-pathway analogue is used for uptake of protons needed for NO reduction (Eq. 1), the reaction would be electrogenic. For *Gs*qNOR, detailed analysis of the catalytic mechanism is hampered by the replacement of the active-site Fe_B_ by a redox inactive zinc^15^. In this study, we therefore established procedures for obtaining a highly active form of the qNOR from *N. meningitidis*, the structural and functional properties of which are discussed below.

The spectroscopic data indicated that heme *b* in *Nm*qNOR adopted a 6-coordinated low-spin configuration, and that heme *b*_3_ in the active site had a mixture of 5- and 6-coordinated structures in the resting state. A mixture of states of heme *b*_3_ was also reported in a qNOR from *Pyrobaculum (P.) aerophilum*^17^. It is plausible that an oxo ligand bridges the non-heme iron Fe_B_ and the heme *b*_3_ iron, the latter of which is then not coordinated by the proximal His. This would form a 5-coordinated high-spin heme as observed in the *P. denitrificans* cNOR^30^. In the 6-coordinated fraction, the heme *b*_3_ iron could be coordinated by both an oxo ligand and the proximal His. The presence of an oxo ligand at the active site in *Nm*qNOR is supported by the observation of substrate inhibition during NO turnover, since recent theoretical work suggested that substrate inhibition could be due to formation of a nitrite ligand by a reaction between the NO substrate and the oxo ligand^31^.

In the reduced state, both hemes *b*_3_ and *b* adopt a 6-coordinated low-spin state in the *Nm*qNOR. In contrast, a 5-coordinated heme *b*_3_ was observed in the reduced *P. denitrificans* *c*NOR^32^, *P. aeruginosa c*NOR^33^ and the *P. aerophiluma* qNOR^17^. Heme *b*_3_ in the reduced state is coordinated by the proximal His, and the 6^th^ coordination site is due to an unknown ligand. However, in the reduced *P. denitrificans c*NOR, there is also evidence for a 6-coordinated heme *b*_3_ population, and that this 6^th^ ligand, possibly a hydroxide, is displaced by exogenous ligand (CO) binding^34^. Presumably in both qNOR and *c*NOR, the 6^th^ ligand is easily dissociated upon NO binding during turnover. Thus, qNOR shares many structural features with *c*NOR.

The catalytic activity observed with *Nm*qNOR is at ~30 e^−^/s/qNOR comparable to those obtained with *c*NORs^10,20,35^, and higher than those previously reported for qNORs^15,17,18^. The activity with MD, the inhibition by HQNO as well as the non-reactivity with the cyt. *c*/TMPD/Asc reduction system supports that the quinol moiety of MD binds specifically to *Nm*qNOR as predicted from the sequence alignment (Fig. S1). Thus quinol compounds, supposedly ubiquinol (since *N. meningitidis* cannot synthesize menaquinone^36^), most likely function as the physiological electron donor. This means that although *N. meningitidis* has a *bc*_1_ complex and several soluble *c* cytochromes (as well as a C-type HCuO), electrons for NO reduction branch off at the quinol level^36^.

As also observed for *c*NOR (see e.g.^20,37^), NO reduction in *Nm*qNOR is substrate-inhibited at high (>20 µM) concentrations of NO, manifested by the sigmoidal behavior (Fig. 4) observed with both the MD/DTT and PMS/Asc electron donor-mediator systems. The maximum activity occurs at 5–10 µM NO, similar to in *c*NOR. Also, the turnover activity of the *Nm*qNOR with O_2_ is, at ~2 e^−^/s/qNOR, comparable to that observed for *c*NOR^22^.

These similarities indicate that the catalytic mechanisms in *c*NOR and qNOR have many features in common. However, the pH dependence of the catalytic reaction is different between *Nm*qNOR and *c*NOR. The activity in *Nm*qNOR (Supplementary Fig. S2) shows a pH optimum at pH 7–7.5 and decreases both at higher and lower pH. This behavior is different from that observed for *c*NORs^14,38^ which shows increasing activity at lower pH, but also different from *Gs*qNOR which shows an increasing activity at higher pH^38^. The differences between the *c*NOR and *Nm*qNOR are presumably linked to differences in proton transfer dynamics whereas the pH dependence in the *Gs*qNOR could be due to rate limitation by other reactions as the overall catalytic activity is low.

Furthermore, the liposome-reconstituted *Nm*qNOR exhibits unique functional properties. Strikingly, NO reduction as catalysed by qNOR-liposomes, using both the MD/DTT and PMS/Asc electron mediator systems, shows a clear respiratory control ratio (RCR) of ~2 (Fig. 5 and Table 1) when a protonophore (CCCP) is added. A similar RCR is observed also upon addition of only VAL, a K^+^ ionophore (Supplementary Table S1). This indicates buildup of a gradient that can be relieved by either dissipation of the ΔpH (CCCP) or the electrical charge only (VAL). Furthermore, the control experiment (Supplementary Fig. S3) with *c*NOR (known to be non-electrogenic^5–7^) shows an RCR of <1 excluding that the electron donor/mediator system is responsible for the RCR observed. We also note that the *Nm*qNOR liposomes show clear substrate inhibition only for uncoupled conditions (Fig. 5). This is consistent with proton uptake being slowed and rate-limiting in the coupled, but not in the uncoupled conditions, supporting that the reaction is electrogenic. We find that the most likely explanation for these observations is that there is a proton electrochemical gradient buildup during NO reduction by *Nm*qNOR.

Recently, a third class of NORs was characterised in terms of electrogenicity; the *B. azotoformans* Cu_A_NOR, which is more closely related to B-type HCuOs^8^ than to the C-type which is the one closest to *c*NOR and qNOR^39^. This Cu_A_NOR shows very high NO-reduction activity^9^, and displays an RCR of 2.4 in liposomes, indicative of an electrogenic reaction^9^. In the Cu_A_NOR study, the electron donor was PES (phenazine ethosulfate), which is very similar to the PMS (phenazine methosulfate) used as a mediator in our studies, so that the discussion by Al-Attar *et al*.^9^ on the possible scenarios for the origin of RCRs in Cu_A_NOR should be relevant also for our studies.

So if NO-reduction *can* be coupled to electrogenic proton transfer as indicated by this study as well as that on Cu_A_NOR, why is this not the case for *c*NOR? One suggested reason, based on theoretical work^40^, is that although the overall process is highly exergonic, the processes (mainly re-reduction of the active site) that are coupled to proton transfer are not, making it very difficult to link them to endergonic and electrogenic proton uptake. This explanation could be consistent with the present study since in qNORs, the quinol electron donors generally have low midpoint potentials and thus the total driving force is higher than in *c*NORs. Such higher driving force in qNOR would lead to a less endergonic reduction, possibly enabling electrogenic proton transfer. However, the total driving force is not very different between the Cu_A_NOR and the *c*NORs, so the electrogenicity observed in Cu_A_NOR does not support the suggestion from the theoretical work, unless the midpoint potential of the active site is significantly different between Cu_A_NOR and *c*NOR.

In Cu_A_NOR as well as in *Nm*qNOR, since we presume that proton uptake is electrogenic, there should thus be proton pathways leading from the cytosolic side up to the active site. In Cu_A_NOR, this pathway is possibly an equivalent of the K-pathway analogue of B-type HCuOs, many features of which are conserved to the Cu_A_NOR. In *Nm*qNOR, the best candidate for such a pathway is an equivalent of the water-filled putative proton pathway, also a K-pathway analogue, leading from the cytosolic side up to the active site observed in the *Gs*qNOR^15^ (see Fig. 7). We used the 4.5 Å resolution structure of *Nm*qNOR (Fig. 6 and overlay with *Gs*qNOR in Fig. 7) to suggest residues possibly participating in a similar pathway, and focused on E-259 at the entrance, E-573 and A-527 at the middle, and E-498 near the active site.

The A-527 variants did not express, while exchanging E-259 and/or E-573 had no or very small effects on catalysis (Table 2). We can consider the possibility that the pathway could indeed be blocked in e.g. the E259L/E573F variant, meaning that proton uptake now occurs from the outside, but fast enough not to be rate-limiting for turnover (i.e. faster than ~30 s^−1^). However, the co-reconstitution experiments (Supplementary Table S3) indicated that there are no significant differences between normalized RCRs in wild-type qNOR and the E259L/E573F variant. Altogether, our data indicate that the same pathway for protons is used in E259L/E573F as in the wild-type, and suggest that neither the E-259 nor the E-573 is crucial for rapid proton uptake.

Contrary to the other variants, exchanging the E-498 residue in *Nm*qNOR to either Ala, Gln or Phe had severe effects on steady-state turnover (Table 2). This is in sharp contrast to the equivalent Ala exchange in *c*NOR (E202A in *P. denitrificans c*NOR, corresponding to E-215 in *P. aeruginosa* (Fig. 7c)), where there are only small effects on turnover^41^ and no effects on the proton-coupled electron transfer characteristics^42^. Although in the E498A and E498Q *Nm*qNOR variants, lowered NO reduction activity could be due to the loss of a significant fraction of the non-heme iron (Table 2), the E498F variant retained the non-heme iron. Thus, a role for the E-498 in proton transfer is still possible. In this context it is interesting to note that the conserved E-512 in *Gs*qNOR^15^, equivalent of the E-494 above E-498 (Fig. 7) in *Nm*qNOR, is, in contrast to the equivalent residue in *c*NOR (E-211 in *P. aeruginosa c*NOR^10^), not a ligand to the non-heme iron Fe_B_. This structural difference might be due to the replacement of Fe_B_ with a Zn in the *Gs*qNOR structure; however, it could also be an indication that there are different functional roles for the E-498 in qNOR versus *c*NOR.

The lack of effects on turnover activity in the E-259 variant, in a similar position to the *Gs*qNOR E-281 residue at the channel entrance (Fig. 7), could be explained if there are redundant pathways at the surface (the E-281 is poorly conserved^24^). The *Gs*qNOR E-591 (E-573 in *Nm*qNOR), further up in the channel, shows 70% conservation in qNORs^24^, but the E573F variant, having a rather drastic change, showed no significant effects and we thus consider it unlikely that protons pass through E-573. On the basis of these observations one could argue that protons, just as in *c*NOR, rather come from the periplasmic solution. In this context, we note that several of the residues important for proton transfer from the periplasm in the *P. denitrificans c*NOR, such as K-54^C^ (NorC), E-58^C^ (NorC) and D-185^14^, are located in the interface between the NorC and NorB subunits, and not conserved to the cyt. *c* -like domain in qNOR^1^. Thus, such a pathway is either not present in qNOR, or composed of an entirely different set of residues, the importance of which we could not test by mutagenesis. The water channel in the *Gs*qNOR structure is rather wide, most residues lining it are poorly conserved, and it is possible that protons can take multiple routes up to around the E-516 (E-498 in *Nm*qNOR). It is clear from our data that the role of the E-498 is different between qNOR and *c*NOR. This Glu shows high conservation in the whole NOR family, which seemed rather puzzling considering the lack of effect of exchanging it in *c*NOR. However, in qNOR it is a crucial residue, an experimental observation that could be used to argue that qNORs were first in evolution, and that this Glu is an evolutionary remnant in *c*NOR.

In summary, we have expressed and purified a highly active qNOR, the *N. meningitidis* enzyme. It shows spectroscopic and kinetic properties that are in many aspects similar to *c*NORs. However, in contrast to *c*NORs, the *Nm*qNOR is an electrogenic enzyme, such that we assume that protons used in catalysis originate in the cytoplasm. Sequence, structural and mutagenesis analysis shows many interesting differences to *c*NOR, but a detailed tracing of the proton pathway responsible for the electrogenic proton uptake will be the subject of future studies. The electrogenicity of qNOR is an important piece for the puzzle of understanding both the evolution of the HCuO superfamily and possible mechanisms of energy conservation. It’s further interesting to note that many pathogenic bacteria utilize qNOR and not *c*NOR for detoxification of NO produced by the hosts’ immune system, which is possibly related to this difference in energy conservation.

## Material and Methods

### Cloning of recombinant *N. meningitidis* qNOR and construction of site-directed variants

A pET-22b vector containing, between the NdeI and XhoI sites, synthetic DNA for *Nm*qNOR, with codons optimized for *E. coli* expression, was purchased from Biomatik (Ontario, Canada). The amplification of the DNA fragment encoding *Nm*qNOR and the introduction of a HindIII site at the C-terminus was carried out by PCR. The DNA fragment was digested by NdeI and HindIII and cloned into a modified pRSET-C plasmid that contains a C-terminal 10 × His-tag immediately after an HRV-3c protease sensitive site (LEVLFQGP).

Site-directed mutations were introduced by QuikChange mutagenesis kit II (Agilent technologies) using the pRSET-C expression vector as a template. The sequences of all expression vectors were confirmed by DNA sequencing. The variants prepared are described in Results.

### Purification of recombinant wild-type and variant *N. meningitidis* qNORs

An *E. coli* strain C43 (DE3) carrying the pRSET-C expression vector for *Nm*qNOR was cultured in 2xYT medium at 37 °C. When the OD_600_ of the culture reached around 0.5, IPTG was added to a final concentration of 0.5 mM to induce expression. After a further ~16 hours growing at 37 °C, the *E. coli* cells were harvested. The cells were suspended in 50 mM Tris-HCl pH 8.0, 150 mM NaCl, and lysed by sonication (Sonifier 250, BRANSON). The membrane fraction was isolated from the lysate by ultracentrifugation (125,000 × g), and membranes, at a total protein concentration of 5–7 mg/mL were solubilized by 1.0% DDM (Dojindo) in 50 mM Tris-HCl pH 8.0, 150 mM NaCl and at 4 °C. The insoluble material was removed by ultracentrifugation (125,000 × g), and the solubilized fraction was applied to a Ni-NTA column (Qiagen) pre-equilibrated with water. The column was washed with 50 mM Tris-HCl pH 8.0, 150 mM NaCl, 0.05% DDM, and 30 mM imidazole, and the enzyme was eluted by 50 mM Tris-HCl pH 8.0, 150 mM NaCl, 0.05% DDM, and 200 mM imidazole. The sample fractions were concentrated by Amicon Ultra 50 K concentrator (Millipore). The concentrated enzyme was applied to a Superdex 200 column (GE Healthcare) pre-equilibrated with 50 mM Tris-HCl pH 8.0, 150 mM NaCl, and 0.1% *n*-decyl-β-D-maltoside (DM; Dojindo). The purified fractions were analyzed by SDS-PAGE (c-Pagel (Atto corporation), 12.5% gel with Tris-glycine running buffer). The samples for SDS-PAGE were not boiled in the SDS loading buffer to avoid aggregation. Purified fractions showing a single band in SDS-PAGE and A_408_/A_280_ greater than 0.7, were employed for further studies. The purity of the variant preparations had an A_408_/A_280_ of ~0.6.

In order to cleave off the His-tag, the PreScission protease (GE Healthcare) was added to the sample eluted from the Ni-NTA column at a ratio of 1 unit protease per 50 μg enzyme, followed by overnight dialysis against 50 mM Tris-HCl pH 8.0, 150 mM NaCl, and 0.05% DDM at 4 °C. The digested enzyme was applied to a Ni-NTA column pre-equilibrated with 50 mM Tris-HCl pH 8.0, 150 mM NaCl, and 0.05% DDM to remove the enzyme retaining His-tag. The flowthrough fractions containing the enzyme were concentrated by Amicon Ultra 50 K concentrator (Millipore), and were further purified by a Superdex 200 column (GE Healthcare) as in the case for the His-tagged sample. The purified enzymes were stored at −80 °C until use. The increase in purity during purification was followed by monitoring the specific NO reduction activity which showed the following values; isolated membranes: 7–10 nmol/s/mg protein, solubilized membranes: ~7 nmol/s/mg protein, purified protein (with and without His-tag): ~300 nmol/s/mg protein (corresponding to ~25 NO/s/qNOR). Thus, the specific activity increased ~30 times during purification.

This preparation is thus highly pure as indicated by the high A_408_/A_280_ ratio, the single band on SDS-page (which at ~70 kDa has very little overlap with common Ni-binding contaminants in *E.coli*) and the good correlation between the activity calculated per mg of total protein to that calculated per *Nm*qNOR molecule (see Results).

The structural and functional properties are essentially the same between *Nm*qNOR with His-tag and without His-tag. Therefore, for the qNOR variants the His-tagged samples were used for further experiments.

The heme content was determined by the pyridine hemochrome method^43^, and this analysis was used to determine the extinction coefficient (ε_408_ (ox) = 213 mM^−1^cm^−1^) of *Nm*qNOR used for all following concentration determinations.

The *Nm*qNOR wild-type and variants used for liposome reconstitution were purified essentially in the same way with the following differences: after induction, *E. coli* C43 (DE3) cells were incubated at 30 °C and the cells were disrupted by passing 2 times through a constant cell disrupter at 35 kpsi (Constant Systems Ltd.). The solubilized enzyme bound to a 5 mL prepacked HisTrap-column (GE Healthcare) was washed with 5 column volumes buffer containing 50 mM imidazole and eluted with an imidazole gradient (50–500 mM imidazole) using an HPLC (Agilent Technologies) in a total elution volume of 50 mL and a flow-rate of 1 mL/min. The qNOR eluted at ~100 mM imidazole was pooled, and washed free of imidazole by repeated concentration and dilution in 50 mM Tris-HCl pH 8.0, 150 mM NaCl, and 0.05% DDM buffer using an Amicon Ultra 100 K concentrator (Millipore). Furthermore, there was no exchange of DDM for DM.

### Analysis of total metal content and non-heme iron content (ferene method)

Metal contents of the purified samples were analyzed by iCE 3400 atomic absorption spectrophotometer (Thermo Fischer Scientific). About 50 μM purified qNOR was diluted 10 times in 60% nitric acid, followed by 100 times dilution in 1% nitric acid. Standard curves were calculated using standard solutions of Fe (0–20 μg/L), Zn (0–5 μg/L) and Cu (0–10 μg/L) in 1% nitric acid. To avoid metal contamination, acid-washed plastic cuvettes were used for the measurements.

Non-heme iron content was assayed as in the reported method^44^, and previously used for *c*NOR^20^. Briefly, 4 µM of qNOR was precipitated by addition of hydrochloric acid. The free non-heme iron was separated from the precipitated protein by centrifugation at 14000 rpm for 15 min and the solution neutralized by addition of ~1.7 M sodium acetate. The non-heme iron was reduced with a small volume of 1 M Asc (pH 5-6) followed by addition of ferene to ~170 µM. The iron content was calculated using ɛ_593nm_ = 35.5 mM^−1^cm^−1^. Background absorbance at 593 nm and iron content of buffer and additions were subtracted.

### UV-Vis absorption spectra

Absorption spectra were recorded on a U-3300 spectrophotometer (Hitachi). The samples for spectroscopic measurements were diluted in 50 mM Tris-HCl pH 8.0, 150 mM NaCl, and 0.1% DM. The reduced qNOR samples were prepared by the addition of an excess amount of dithionite to the oxidized enzyme under N_2_ atmosphere.

### Resonance Raman spectra

Resonance Raman spectra were obtained with a liquid nitrogen-cooled CCD detector (Roper Scientific, Spec 10:400B/LN) attached to a single polychrometer (Jobin Yvon, SPEX750M). The 413.1-nm line from a Kr ion laser (Spectra Physics, model BeamLok 2060) was utilized as excitation source. The laser power at the sample point was adjusted to 0.1~1 mW. Raman shifts were calibrated using indene, acetone and saturated aqueous solution of ferrocyanide. The measurements were carried out with a quartz spinning cell to avoid local heating. The qNOR concentration was adjusted to 20–40 μM in 50 mM Tris-HCl pH 8.0, 150 mM NaCl, and 0.1% DM. The reduced form was prepared the same way as for the optical absorption measurements.

### Crystallization and X-ray crystal structure analysis

For the crystallization, the concentrated sample without His-tag was applied to a Superdex 200 column (GE Healthcare) pre-equilibrated with 50 mM Tris-HCl pH 8.0, 150 mM NaCl, and 0.5% *n*-decyl-β-D-thiomaltoside (DTM; Anatrace). DTM-solubilized *N. meningitidis* qNOR was initially crystallized at 20 °C by sitting drop vapor diffusion method in drops containing ~15 mg/mL solution (1 μL) mixed with an equal volume of a reservoir solution of 27% (v/v) PEG 400, 1 mM CdCl_2_, 25 mM MgCl_2_, 0.1 M MES pH 6.5. The crystals with ~30 μm in size were obtained and crushed for using as microseeds. The crystals for the X-ray diffraction experiments were obtained at 10 °C by sitting drop vapor diffusion with microseeding method in drops containing ~15 mg/mL solution (1 μL) mixed with an equal volume of a reservoir solution of 22% (v/v) PEG 350 monomethyl ether, 100 mM MgCl_2_, 10 mM Urea, 10% glycerol. Red-colored crystals, ~100 × 100 × 50 μm^3^ in size were obtained within a few days. The crystals were flash-frozen by liquid nitrogen.

X-ray diffraction data were collected at 100 K at a wavelength of 1.0 Å on beamline BL41XU at SPring-8 using a MX225HE CCD detector (Rayonix). The data were integrated using AutoPROC^45,46^ and scaled and merged in Aimless^47^. Data collection statistics are summarized in Table S2. An initial model was obtained by molecular replacement using Phaser of PHENIX suite^48^ with the crystal structure of *G. stearothermophilus* qNOR (PDB ID: 3AYF) as a search model. The refinement of the low resolution structure (4.5 Å resolution) was performed using REFMAC^49^. Close agreement between the electron density and the arrangement of transmembrane helices (*C*α trace) was confirmed by COOT. The statistics for the refinement are summarized in Table S2.

### NO and O_2_ reduction activity measurements

NO reduction was measured at room temperature in a total volume of 1 mL 20 mM K^+^-HEPES (pH 7.4), 100 mM KCl, 0.05% DDM (if not indicated otherwise) using a NO sensor (World Precision Instruments). The solution was made anaerobic by incubation in 10 mM glucose, 100 U/mL catalase and 10 U/mL glucose oxidase for at least 10 min. This procedure was sufficient to reach essentially fully anaerobic conditions, as previously measured with an O_2_-electrode (Hansatech). Electron donors and mediators (final concentrations: 1 mM MD/5 mM DTT or 10 µM PMS/6 mM Asc or 10 µM horse heart cytochrome *c* (cyt. *c*)/0.5 mM TMPD/6 mM Asc) were supplied followed by stepwise addition of 5 × 10 µM NO from a saturated NO solution (2 mM). To start the reaction qNOR or *c*NOR (10–40 nM) was added and the maximum rate calculated from at least 3 measurements performed on the same day. Due to the substrate inhibition displayed by qNOR, rates of NO consumption were calculated from the maximal slopes obtained at ~5 µM NO. NO consumption rates were also studied in the presence of the quinol analogue HQNO (2-heptyl-4-hydroxyquinoline N-oxide) which inhibits qNOR.

For the comparisons of activity in DDM and DM, and the measurements of the PMS/Asc activities in the variants, NO consumption was monitored in essentially the same way, with the following differences: measurements were performed at 20 °C, the assay solution contained 50 mM HEPES pH 7.0 and 0.05% DDM. The oxygen scavenging system consisted of 10 mM D-glucose, 2 U/mL glucose oxidase and 40 U/mL catalase. NO was added to a final concentration of 20 μM.

Oxygen reduction was measured in a total volume of 1 mL 20 mM K^+^-Hepes (pH 7.4), 100 mM KCl, 0.05% DDM using a Clark-type electrode (Hansatech). Electron donors and mediators (1 mM MD/5 mM DTT) were supplied to air-saturated buffer. To start the reaction ~350 nM qNOR was added after background monitoring. The maximum rate was calculated from at least 3 measurements.

### Reconstitution into liposomes and determination of respiratory control ratios

Small unilamellar liposomes (SUV) were prepared by sonication of 50 mg/mL purified soybean lipids. 4 µM qNOR was incubated with 1.5% sodium cholate and the pre-formed liposomes for 1 h at RT with gentle shaking. Removal of cholate was achieved by gel-filtration using a PD-10 column (GE Healthcare). Under these conditions, there is on average less than one enzyme/liposome (i.e. many liposomes are empty). The proteoliposomes were further concentrated to the initial volume using an Amicon Ultra 100 K concentrator (Millipore). The reconstitution efficiency was calculated using reduced minus oxidized static difference spectra of qNOR in the proteoliposomes.

NO reduction by liposome-reconstituted qNOR was measured as described above. Respiratory control ratios (RCRs) were calculated as the ratio of the uncoupled (+CCCP) and coupled (-CCCP) maximum rate of at least 3 measurements, and the standard deviations given in Table 2 is between sets of experiments. Comparison between wild-type and E259L/E573F variant qNOR were performed side-by-side on the same day under the same conditions. Control experiments adding CCCP and VAL to detergent-solubilised qNOR showed no impact (for CCCP) or showed inhibition of the NO-reduction activity (for VAL, to 65–75% of original activity). The inhibition observed with VAL means that the increase observed in liposomes (see Results) is presumably somewhat underestimated.

Note that we did not attempt to measure proton pumping, i.e., acidification on the outside of liposomes, as measurements of small pH changes in unbuffered solutions are very difficult to perform in the presence of NO.

### Co-reconstitution of qNOR and cytochrome *c* oxidase into liposomes

In order to compensate/control for possible differences in the quality and tightness of the liposomes, we co-reconstituted qNOR with the A-type HCuO (cyt. *aa*_3_) from *Rhodobacter (R.) sphaeroides*. In this way, the RCR for O_2_ reduction by the cyt. *aa*_3_ with the electron donor-mediator-system 20 µM cyt. *c*/0.5 mM TMPD/6 mM Asc can be directly compared to the RCR for NO reduction (catalyzed solely by qNOR as cyt. *aa*_3_ has no NO reduction activity^50^) with the electron donor-mediator-system 1 mM/5 mM DTT and in the same liposomes. Co-reconstitution was achieved by the same method as ‘normal’ reconstitution except that both 2 µM qNOR and 2 µM *aa*_3_ were added to the preformed liposomes. This means that each liposome is either empty, contains qNOR, or contains *aa*_3_. The purified *aa*_3_ oxidase for co-reconstitution experiments was obtained from *R. sphaeroides* as previously described^51,52^.

### Data availability

Coordinates and structure factors from the structural determination have been deposited in the Protein Data Bank with accession codes 6ELH and 6EHL-sf, respectively.

## Electronic supplementary material

Supplementary File

## References

[1] Tosha T, Shiro Y (2013). Crystal structures of nitric oxide reductases provide key insights into functional conversion of respiratory enzymes. IUBMB Life.

[2] Pereira MM, Santana M, Teixeira M (2001). A novel scenario for the evolution of haem-copper oxygen reductases. Biochim. Biophys. Acta.

[3] Lee HJ, Reimann J, Huang Y, Ädelroth P (2012). Functional proton transfer pathways in the heme-copper oxidase superfamily. Biochim. Biophys. Acta.

[4] Sharma V, Wikström M (2016). The role of the K-channel and the active-site tyrosine in the catalytic mechanism of cytochrome c oxidase. Biochim. Biophys. Acta.

[5] Bell LC, Richardson DJ, Ferguson SJ (1992). Identification of nitric oxide reductase activity in *Rhodobacter capsulatus*: the electron transport pathway can either use or bypass both cytochrome c2 and the cytochrome bc1 complex. J. Gen. Microbiol..

[6] Hendriks JH, Jasaitis A, Saraste M, Verkhovsky MI (2002). Proton and electron pathways in the bacterial nitric oxide reductase. Biochemistry.

[7] Reimann J, Flock U, Lepp H, Honigmann A, Ädelroth P (2007). A pathway for protons in nitric oxide reductase from *Paracoccus denitrificans*. Biochim. Biophys. Acta.

[8] Heylen K, Keltjens J (2012). Redundancy and modularity in membrane-associated dissimilatory nitrate reduction in Bacillus. Front. Microbiol..

[9] Al-Attar S, de Vries S (2015). An electrogenic nitric oxide reductase. FEBS Lett.

[10] Hino T (2010). Structural basis of biological N2O generation by bacterial nitric oxide reductase. Science.

[11] Thorndycroft FH, Butland G, Richardson DJ, Watmough NJ (2007). A new assay for nitric oxide reductase reveals two conserved glutamate residues form the entrance to a proton-conducting channel in the bacterial enzyme. Biochem. J..

[12] Flock U (2008). Defining the Proton Entry Point in the Bacterial Respiratory Nitric-oxide Reductase. J. Biol. Chem..

[13] ter Beek J, Krause N, Reimann J, Lachmann P, Ädelroth P (2013). The nitric-oxide reductase from Paracoccus denitrificans uses a single specific proton pathway. J. Biol. Chem..

[14] ter Beek J, Krause N, Ädelroth P (2016). Investigating the Proton Donor in the NO Reductase from *Paracoccus denitrificans*. PLoS One.

[15] Matsumoto Y (2012). Crystal structure of quinol-dependent nitric oxide reductase from Geobacillus stearothermophilus. Nat. Struct. Mol. Biol..

[16] Stevanin TM, Moir JW, Read RC (2005). Nitric oxide detoxification systems enhance survival of *Neisseria meningitidis* in human macrophages and in nasopharyngeal mucosa. Infect. Immun..

[17] de Vries S, Strampraad MJF, Lu S, Moënne-Loccoz P, Schröder I (2003). Purification and characterization of the MQH(2): NO oxidoreductase from the hyperthermophilic archaeon *Pyrobaculum aerophilum*. J. Biol. Chem..

[18] Cramm R, Pohlmann A, Friedrich B (1999). Purification and characterization of the single-component nitric oxide reductase from *Ralstonia eutropha* H16. FEBS Lett.

[19] Lou BS (2000). Resonance Raman studies indicate a unique heme active site in prostaglandin H synthase. Biochemistry.

[20] Girsch P, deVries S (1997). Purification and initial kinetic and spectroscopic characterization of NO reductase from *Paracoccus denitrificans*. Biochim. Biophys. Acta.

[21] Koutny M, Kucera I (1999). Kinetic analysis of substrate inhibition in nitric oxide reductase of *Paracoccus denitrificans*. Biochem. Biophys. Res. Commun..

[22] Flock U, Watmough NJ, Ädelroth P (2005). Electron/Proton Coupling in Bacterial Nitric Oxide Reductase during Reduction of Oxygen. Biochemistry.

[23] Salomonsson L (2012). Proton transfer in the quinol-dependent nitric oxide reductase from *Geobacillus stearothermophilus* during reduction of oxygen. Biochim. Biophys. Acta.

[24] Sheradan, P. Characterization of the quinol nitric oxide reductase from *Persephonella Marina*. *Thesis, University of Illinois-Urbana Champaign* (2013).

[25] Saraste M, Castresana J (1994). Cytochrome oxidase evolved by tinkering with denitrification enzymes. FEBS Lett..

[26] Gribaldo S, Talla E, Brochier-Armanet C (2009). Evolution of the haem copper oxidases superfamily: a rooting tale. Trends Biochem. Sci..

[27] Ducluzeau AL (2014). The evolution of respiratory O2/NO reductases: an out-of-the-phylogenetic-box perspective. J. R. Soc. Interface.

[28] Hemp J (2007). Comparative Genomics and Site-Directed Mutagenesis Support the Existence of Only One Input Channel for Protons in the C-Family (cbb(3) Oxidase) of Heme-Copper Oxygen Reductases. Biochemistry.

[29] Chang HY, Hemp J, Chen Y, Fee JA, Gennis RB (2009). The cytochrome ba3 oxygen reductase from *Thermus thermophilus* uses a single input channel for proton delivery to the active site and for proton pumping. Proc. Natl. Acad. Sci. USA.

[30] Pinakoulaki E, Gemeinhardt S, Saraste M, Varotsis C (2002). Nitric-oxide reductase. Structure and properties of the catalytic site from resonance Raman scattering. J. Biol. Chem..

[31] Blomberg MR, Siegbahn PE (2012). Mechanism for N(2)O generation in bacterial nitric oxide reductase: a quantum chemical study. Biochemistry.

[32] Moënne-Loccoz P, de Vries S (1998). Structural characterization of the catalytic high-spin heme b of nitric oxide reductase: A resonance Raman study. J. Am. Chem. Soc..

[33] Sato N (2014). Structures of reduced and ligand-bound nitric oxide reductase provide insights into functional differences in respiratory enzymes. Proteins.

[34] Hendriks JH (2001). Reaction of carbon monoxide with the reduced active site of bacterial nitric oxide reductase. Biochemistry.

[35] Zumft WG, Braun C, Cuypers H (1994). Nitric oxide reductase from *Pseudomonas stutzeri*. Primary structure and gene organization of a novel bacterial cytochrome bc complex. Eur. J. Biochem..

[36] Rock JD, Moir JW (2005). Microaerobic denitrification in *Neisseria meningitidis*. Biochem. Soc. Trans..

[37] Lachmann P, Huang Y, Reimann J, Flock U, Ädelroth P (2010). Substrate control of internal electron transfer in bacterial nitric-oxide reductase. J. Biol. Chem..

[38] Terasaka E (2014). Characterization of quinol-dependent nitric oxide reductase from Geobacillus stearothermophilus: enzymatic activity and active site structure. Biochim. Biophys. Acta.

[39] Sousa FL, Alves RJ, Pereira-Leal JB, Teixeira M, Pereira MM (2011). A bioinformatics classifier and database for heme-copper oxygen reductases. PLoS One.

[40] Blomberg MR, Siegbahn PE (2013). Why is the reduction of NO in cytochrome c dependent nitric oxide reductase (cNOR) not electrogenic?. Biochim. Biophys. Acta.

[41] Butland G, Spiro S, Watmough NJ, Richardson DJ (2001). Two conserved glutamates in the bacterial nitric oxide reductase are essential for activity but not assembly of the enzyme. J. Bacteriol..

[42] Flock U, Lachmann P, Reimann J, Watmough NJ, Ädelroth P (2009). Exploring the terminal region of the proton pathway in the bacterial nitric oxide reductase. J. Inorg. Biochem..

[43] Berry EA, Trumpower BL (1987). Simultaneous determination of hemes a, b, and c from pyridine hemochrome spectra. Anal. Biochem..

[44] Hennessy DJ, Reid GR, Smith FE, Thompson SL (1984). Ferene - a New Spectrophotometric Reagent for Iron. Can. J. Chem..

[45] Vonrhein C (2011). Data processing and analysis with the autoPROC toolbox. Acta Crystallogr D Biol Crystallogr.

[46] Kabsch W (2010). Xds. Acta Crystallogr D Biol Crystallogr.

[47] Evans PR, Murshudov GN (2013). How good are my data and what is the resolution?. Acta Crystallogr D Biol Crystallogr.

[48] McCoy AJ (2007). Phaser crystallographic software. J Appl Crystallogr.

[49] Murshudov GN (2011). REFMAC5 for the refinement of macromolecular crystal structures. Acta Crystallogr D Biol Crystallogr.

[50] Stubauer G, Giuffre A, Brunori M, Sarti P (1998). Cytochrome c oxidase does not catalyze the anaerobic reduction of NO. Biochem. Biophys. Res. Commun..

[51] Vilhjalmsdottir J, Johansson AL, Brzezinski P (2015). Structural Changes and Proton Transfer in Cytochrome c Oxidase. Sci. Rep..

[52] Zhen Y (1998). Overexpression and purification of cytochrome c oxidase from Rhodobacter sphaeroides. Protein Expr. Purif..

