# Supplementary material for: Characterization of the quinol-dependent nitric oxide reductase from the pathogen Neisseria meningitidis, an electrogenic enzyme
Source: Sci Rep. 2018 Feb 26;8:3637. doi: 10.1038/s41598-018-21804-0 (PMC5826923; doi:10.1038/s41598-018-21804-0)
Supplement: Supplementary file 1 — Supplementary File [file 41598_2018_21804_MOESM1_ESM.pdf]

Supplementary Information for

Characterization of the quinol-dependent nitric oxide reductase from the pathogen *Neisseria meningitidis*, an electrogenic enzyme.

Nathalie Gonska[1#], David Young[2, 3,4#], Riki Yuki[5], Takuya Okamoto[5], Tamao Hisano[2], Svetlana Antonyuk[3], S. Samar Hasnain[3], Kazumasa Muramoto[5], Yoshitsugu Shiro[2, 5*], Takehiko Tosha[2*], Pia Ädelroth[1*]

[1]Department of Biochemistry and Biophysics, Stockholm University, Svante Arrhenius väg 16C, 10691 Stockholm, Sweden

[2]RIKEN SPring-8 Center, 1-1-1 Kouto, Sayo, Hyogo 679-5148, Japan

[3]Institute of Integrative Biology, Faculty of Health and Life Sciences, University of Liverpool, Liverpool L69 7ZB, UK

[4]Current address: Center for Structural Biology, VIB, B-1050, Brussels, Belgium

[5]Graduate School of Life Science, University of Hyogo, 3-2-1 Kouto, Kamigori, Ako, Hyogo 678-1297, Japan

#equal contribution

*corresponding authors : E-mail: pia.adelroth@dbb.su.se, ttosha@spring8.or.jp, yshiro@sci.u-hyogo.ac.jp

Table S1: NO-reduction by liposome-reconstituted *N. meningitidis* qNOR (*Nm*qNOR): Comparison of RCRs obtained with valinomycin and CCCP. Numbers given are the averages and standard deviations for *n* measurements. Conditions: 20 mM K^+-HEPES (pH 7.4), 100 mM KCl, 10 mM glucose, 100 U/ml catalase, 10 U/ml glucose oxidase, ~40 nM qNOR in liposomes, 1 mM MD/5 mM DTT or10 µM PMS/6 mM Asc, 10 µM CCCP, 10 µM Val as indicated. The RCR with Val was normalized to that obtained with CCCP in the same liposomes.

	**MD/DTT**	**PMS/Asc**
**RCR (CCCP)**	100% (~3)	100%
**RCR (Val)**	80 ± 20% (*n*=3)	82 ± 6% (*n*=3)

Table S2: X-ray data collection and refinement statistics

**Data collection**	
Space group	$P2_12_12_1$
Cell dimensions	
a, *b*, *c* (Å)	93.68, 123.1, 130.85
α, β, γ (°)	90
Resolution (Å)	53.64 – 4.5 (5.03 - 4.5)
R_{meas}	0.203 (3.692)
R_{merge}	0.195 (3.565)
$I / \sigma I$	6.7 (1.0)
Completeness (%)	99.8 (99.9)
Redundancy	14.3 (14.8)
Wilson plot B factor ($Å^2$)	177.9
**Refinement**	
Resolution (Å)	53.64 – 4.5 (4.85 - 4.5)
No. reflections	9388
R_{work} / R_{free} (%)	32.36 / 35.92
No. atoms	
Protein	5975
Ligand/ion	86
Water	0
Atomic displacement parameters ($Å^2$)	
Protein A/B/C	357
Ligand/ion	189
Water	-
R.m.s deviation from target stereochemistry	
Bond lengths (Å)	0.01
Bond angles (°)	1.11

Table S3. Respiratory control ratios (RCR) for NO and O_2-reduction in liposomes with wild-type and the E259L/E573F variant *Nm*qNORs co-reconstituted with cyt. aa_3. Shown is the average and standard deviation for *n* measurements as specified in Materials and Methods. Conditions: 20 mM HEPES (pH 7.4), 100 mM KCl and, for NO measurements, 10 mM glucose, 100 U/mL catalase, 10 U/mL glucose oxidase, 1 mM MD/5 mM DTT, and 50 µM NO. For O_2: 20 µM cyt. *c*/0.5 mM TMPD/6 mM Asc, ~250 µM O_2.

	**RCR (NO)**	**RCR (O_2)**	**RCR_{NO}/RCR_{O2}**
**WT(qNOR)-aa_3**	2.0 ± 0.3 (*n*=2)	3.8 ± 0.3 (*n*=2)	0.5-0.6
**E259L/E573F(qNOR)-aa_3**	1.4 ± 0.1 (*n*=2)	2.6 ± 0.7 (*n*=2)	0.5-0.6

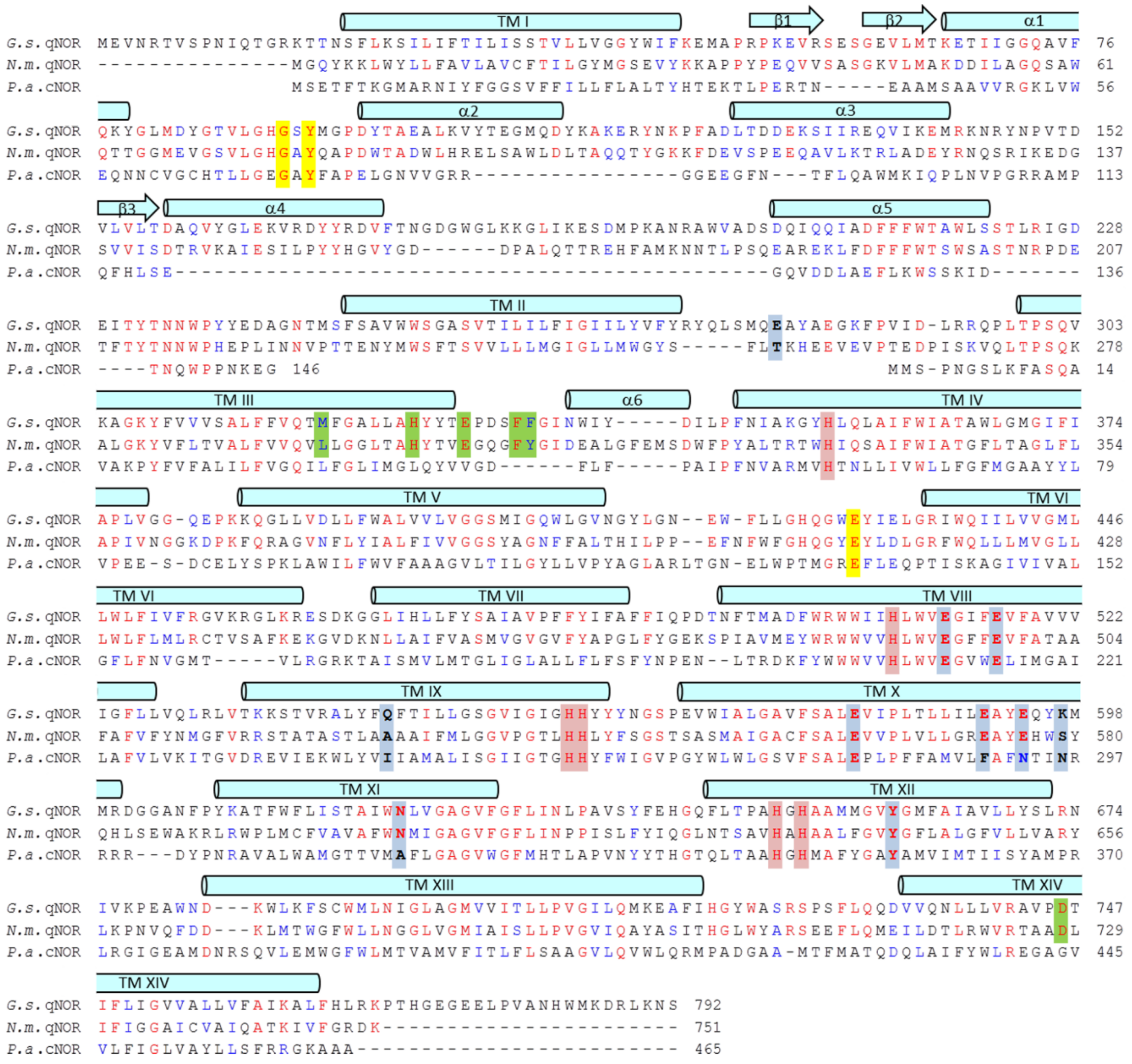

Figure S1. Comparison of amino acid sequence of *N. meningitidis* (*N.m.*) qNOR with those of *G. staerothermophilus* (*G.s.*) qNOR and *P. aeruginosa* (*P.a.*) *c*NOR. The red, blue and black residues indicate conserved, similar and non-conserved residues, respectively. On the basis of the structure of *G. staerothermophilus* qNOR, structurally and functionally important residues are highlighted: pink indicates metal ligands; blue with bold indicates residues located at the suggested water/proton channel; green indicates the HQNO binding site, yellow indicates Ca^{2+} ligands. Cylinders and arrows above the alignment represent α-helices and β-sheets, respectively. The alignment was produced by ClustalX. The calculated mass of the *N. meningitidis* qNOR is 84.2 kD.

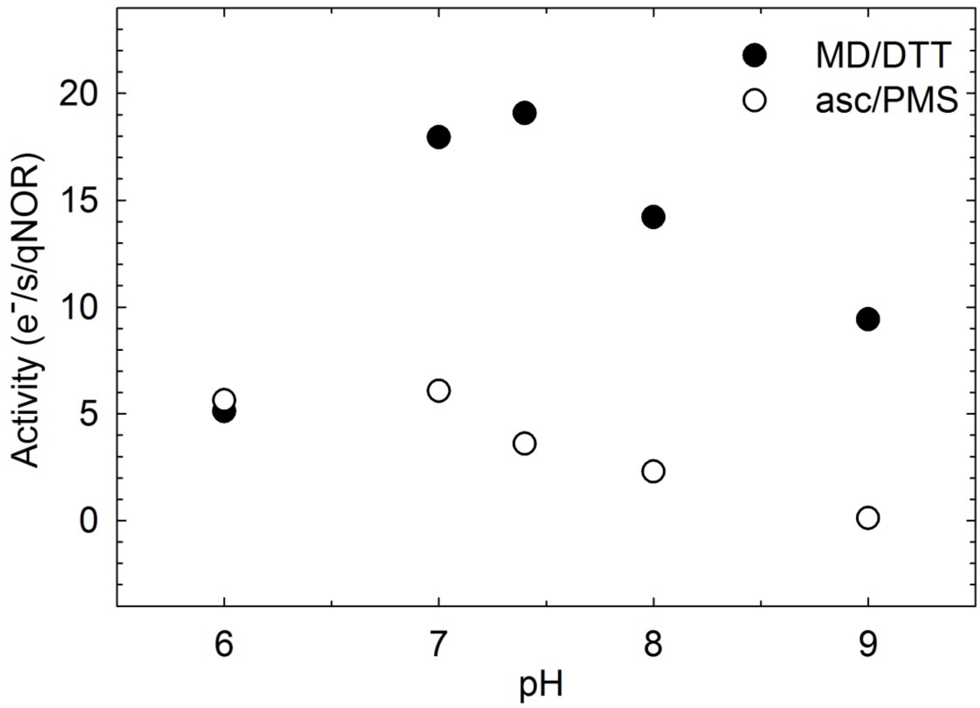

Figure S2: pH-dependence of NO reduction rates catalysed by *Nm*qNOR. Conditions: buffers used depending on pH: 50 mM MES (pH 6), 50 mM MOPS (pH 7), 20 mM HEPES (pH 7.4), 50 mM Tris-HCl (pH 8), or 50 mM CHES (pH 9), 100 mM KCl, 10 mM glucose, 100 U/ml catalase, 10 U/ml glucose oxidase, 50 µM NO, 70 nM qNOR; filled circles: 1 mM MD/5 mM DTT; empty circles: 10 µM PMS/6 mM Asc.

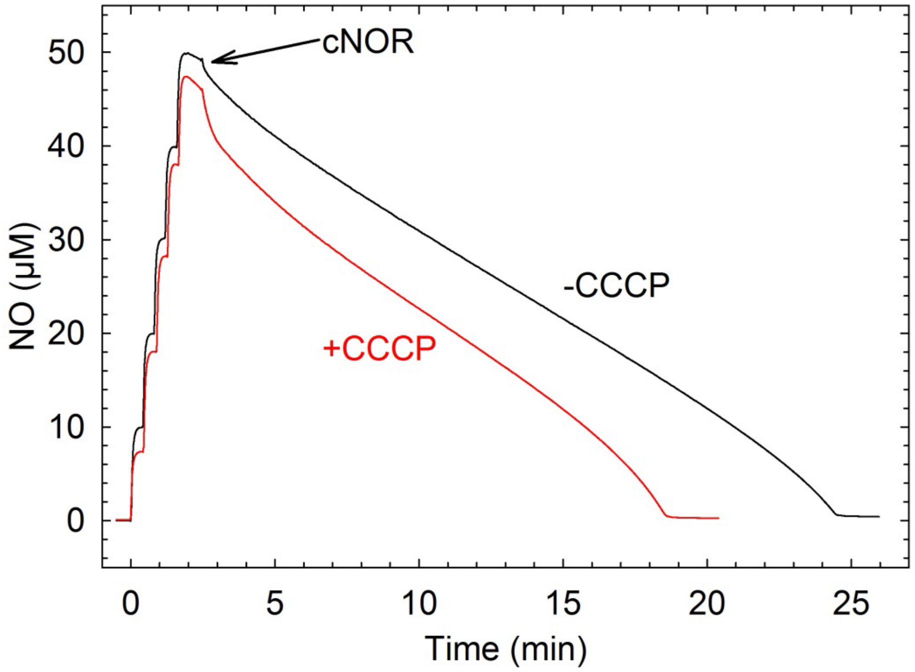

Figure S3: The effect of uncouplers on the activity of *c*NOR in liposomes. Conditions: 50 mM MOPS (pH 7.0), 100 mM KCl, 10 mM glucose, 100 U/ml catalase, 10 U/ml glucose oxidase, 6 mM Asc, 10 µM PMS, (10 µM CCCP for red traces), 40 nM *P. denitrificans* *c*NOR. NO was added in 5 consecutive steps (total of 50 µM); all additions except *c*NOR-liposomes (added where indicated) were made before NO addition. Note that the background NO consumption rate (before *c*NOR addition) increases somewhat in the presence of CCCP but the maximum catalysed rate (at ~5 µM NO) is unaffected by CCCP addition.
